# Robust and sensitive ELISA detection of total and activated PRKN

**DOI:** 10.1080/15548627.2026.2694658

**Published:** 2026-07-03

**Authors:** Jens O. Watzlawik, Bernardo A. Bustillos, Claudia Rodriguez Martinez, Dennis W. Dickson, Zbigniew K. Wszolek, Owen A. Ross, Wolfdieter Springer, Fabienne C. Fiesel

**Affiliations:** a Department of Neuroscience, Mayo Clinic, Jacksonville, FL, USA; b Mayo Graduate School of Biomedical Sciences, Mayo Clinic, Jacksonville, FL, USA; c Department of Neurology, Mayo Clinic, Jacksonville, FL, USA

**Keywords:** Mitochondria, mitophagy, parkin, Parkinson disease, PINK1, ubiquitin

## Abstract

Parkinson disease (PD) is closely linked to disruptions in mitochondrial quality control, a process regulated by the ubiquitin kinase PINK1 and the E3 ubiquitin ligase PRKN/parkin. Upon mitochondrial damage, PINK1 phosphorylates ubiquitin, which in turn recruits and activates PRKN. Full activation of PRKN is mediated by PINK1-dependent phosphorylation of PRKN at serine 65, which leads to widespread ubiquitination of mitochondrial substrates and amplifies the mitophagy response. Disruption of this pathway results in mitochondrial accumulation, oxidative stress, and neuronal death, all key mechanisms of PD pathogenesis. Genetic studies have shown biallelic loss-of-function mutations in PRKN are the most common cause of early-onset PD. Although the role of haploinsufficiency remains under investigation, PRKN protein becomes insoluble and inactive with aging or post-translational modifications, indicating that functional protein levels are a key determinant of disease risk. Reliable quantification of total and activated PRKN in samples has not been feasible, limiting research and clinical assessment. To address this, we developed and validated knockout (KO)-verified sandwich ELISA assays that quantify both total PRKN and PINK1-phosphorylated p-S65-PRKN. These assays provide absolute quantification of PRKN, improving functional diagnosis, and patient stratification in PD. Application of these methods established the concentration of PRKN in cells and in brain and revealed significant effects of a common genetic PRKN variant, further highlighting the importance of determining functional PRKN protein levels. The developed immunoassays complement previously established PINK1 and p-S65-Ub measurements, enhancing mechanistic insight into mitophagy and enabling effective monitoring of PD therapies and other neurodegenerative diseases.

**Abbreviations:** AD: Alzheimer disease; CCCP: carbonyl cyanide 3-chlorophenylhydrazone; ECL: electrochemiluminescence; ELISA: enzyme-linked immunosorbent assay; IBR: In-between-RING; iPSC: induced pluripotent stem cell; KO: knockout; LoB: limit of blank; LoD: limit of detection; LoQ: limit of quantification; MSD: Meso Scale Discovery; PD: Parkinson disease; p-S65-PRKN: serine 65 phosphorylated PRKN; p-S65-Ub: serine 65 phosphorylated ubiquitin; REP: repressor element of PRKN; RING: really interesting new gene; Ub: ubiquitin; UBL: ubiquitin-like domain; VCL: vinculin; WT: wild-type

## Introduction

The ubiquitin (Ub) kinase PINK1 and the E3 Ub ligase PRKN/parkin perform a critical mitochondrial quality control to maintain a pool of healthy organelles (reviewed in [1]). Upon mitochondrial damage, PINK1 stabilizes on the outer mitochondrial membrane, dimerizes and phosphorylates ubiquitin at serine 65 (p-S65-Ub), which recruits PRKN from the cytosol [2–4]. The subsequent PINK1-dependent phosphorylation of PRKN at a conserved serine 65 in its Ub-like (UBL) domain is needed for full activation of its E3 Ub ligase functions [5–7]. Once activated, PRKN ubiquitinates a multitude of substrate proteins [8] and together with PINK1 leads to an amplification of the p-S65-Ub signal on mitochondria. This label is thought to recruit autophagy receptors to feed damaged mitochondria into the autophagosome/lysosome machinery for degradation. Failure to engage this pathway has been shown to lead to increased mitochondrial damage over time which can trigger inflammation and cell death [9,10].

Both *PINK1* and *PRKN* genes are linked to early-onset Parkinson disease (PD), with bi-allelic *PRKN* loss-of-function mutations being the most common genetic cause [11,12]. Although frequently found in the population, the impact of heterozygous mutations is still under debate [13,14] but requires carefully assessment especially as more recent studies revealed the presence of cryptic, copy number variants on the other allele [15]. Genotype-phenotype correlations also highlighted clear dosage effects with greater pathogenicity of *PRKN* structural variants compared to missense mutations [16]. Besides the contribution to familial PD, it has been suggested that PRKN may also play a prominent role in sporadic disease [17]. PRKN protein becomes insoluble during aging and may be inactivated in sporadic PD as a consequence of post-translational modifications of PRKN that have been identified to occur in the human brain [18–24]. Together this indicates that levels of functional PRKN protein are an important measure to assess PD risk beyond just the genetic status of an individual.

The E3 Ub ligase PRKN features a N-terminal Ub-like (UBL) domain followed by a linker, RING0, RING1, In-between-RING (IBR), repressor element (REP), and RING2 domain. In its inactive autoinhibited state, PRKN protein is folded back onto itself in a compact non-accessible structure [25–28]. During activation, PRKN protein undergoes significant structural changes that expose essential domains for binding to its coenzymes and catalytic activity toward Ub, enabling efficient and broad substrate and auto-ubiquitination [29–31], consistent with a rapid decline of its protein during this dynamic sequence [32–34]. Of note, PRKN protein levels are increased in absence of PINK1 in human cells and rodent brain, while structural de-repression of endogenous PRKN or acute activation of the pathway through mitochondrial depolarization rapidly depletes PRKN protein levels [32–34]. As such PRKN protein levels can fluctuate depending on stress-induced activation and cellular turnover rates of damaged mitochondria as (activated) PRKN seems to be consumed during the process potentially as a mechanism to self-regulate [35]. However, the exact levels of physiological PRKN that are needed to fulfill a critical mitophagy function remain undetermined also because currently it is not possible to quantitate levels of total or activated PRKN reliably and sensitively.

Here we developed novel knockout (KO) validated sandwich ELISAs to assess levels of both total PRKN as well as PINK1-phosphorylated p-S65-PRKN from cells and brain tissues. The assays allow absolute quantification of in-/active PRKN and are expected to critically improve diagnosis, prognosis, and stratification of patients beyond genetic risk assessment. Applying those assays, we identified an unexpected, relatively large effect size of common genetic variation in PRKN that has been considered benign, which now warrants further investigation. The outcome of our study suggests that therapeutically relevant PRKN protein levels not only exist, but should be experimentally determined, evaluated, and then targeted if possible. The assays further dovetail with previously established readouts for PINK1 and p-S65-Ub and will deepen the mechanistic understanding of mitophagy and facilitate monitoring of therapeutic modulation in PD and other neurodegenerative diseases.

## RESULTS

### PRKN antibody assessment in western blots using human cell and mouse brain lysates

In order to develop a specific and robust ELISA for the detection of total PRKN, we selected 15 different commercially available antibodies from four host species with most derived from either rabbit or mouse (Table 1). Nine out of 15 antibodies were monoclonal and were raised against either the N (UBL and linker domain) or C terminus (REP and RING2 domain) of PRKN, respectively (Fig. S1A, B). Given the overall high homology (Fig. S1C), most antibodies were predicted to recognize both human and mouse PRKN protein.

**Table 1.** List of used PRKN antibodies for ELISA development.

ID	species	vendor	catalog #	clone	RRID	epitope/antigen
total PRKN antibodies:						
#1	rabbit	Invitrogen	702785	21H24L9	AB_2724937	N terminus aa 1–95^‡^
#2	sheep	MRC PPU Dundee^$^	DU_37370	n/a	AB_3712777	N terminus aa 1–108/GST-PARKIN
#3	rabbit	Cell Signaling Technology	2132	n/a	AB_10693040	residues surrounding aa 400
#4	rabbit	Cell Signaling Technology	32833	D4Z1W	AB_3073958	C terminus
#5	rabbit	Invitrogen	711820	21HCLC	AB_2716914	N terminus aa 1–95^‡^
#6	goat	Novus Biologicals	NB100-53798	n/a	AB_829898	aa 394–409
#7	rabbit	Proteintech	14060–1-AP	n/a	AB_2878005	aa 81–387
#8*	rabbit	Abcam	ab179812	EPR5024(N)		aa 1–100
#9	goat	Santa Cruz Biotechnology	sc-13279	n/a	AB_653853	N terminus
#10	rabbit	RayBiotech	102–16768	n/a	AB_3712776	aa 111–140
#11	mouse	Millipore	MAB5512	PRK8	AB_2267915	aa 400–408/full-length protein
#12	mouse	Invitrogen	39–0900	PRK109	AB_2533396	aa 409–420/full-length protein
#13	mouse	BioLegend	865602	5C3	AB_2810778	N-terminus aa 1–95^‡^
#14	mouse	BioLegend	870501	A15165D	AB_2820194	aa 397–404/full-length protein
#15	mouse	BioLegend	870402	A15165E	AB_2820193	aa 177–184, 443–453, 459–464/full-length protein
p-S65-PRKN antibodies:						
#A	rabbit	Abcam	ab315377	MJF-R17-42–4	AB_3661828	surrounding aa 65
#B^&^	rabbit	MJFF	MJF33-80	n/a		surrounding aa 65
#C^&^	rabbit	MJFF	MJF33-95	n/a		surrounding aa 65
#D*	rabbit	Abcam	ab154995	n/a	AB_2732915	surrounding aa 65
#E	rabbit	Affinity Bioscience	AF3500	n/a	AB_2846814	surrounding aa 65

The epitope/antigen information is based on the vendor datasheet or website, except for where noted. ^$^MRC Protein Phosphorylation and Ubiquitylation Unit Regents and Services, University of Dundee. *Antibody has been discontinued. ^&^Antibodies were obtained from the MJFF and are currently not commercially available. ^‡^This epitope for antibody #1, #5, and #13 is based on the western blot analysis with *prkn*^−/−^ mouse brain. n/a: not applicable.

We first tested the specificity of all selected antibodies on western blots using either WT or *PRKN* knockout (KO) human cell lysates and mouse brain lysates, respectively (Figure 1). As an additional control, we included *PINK1* KO samples, which were previously shown to have increased PRKN protein levels due to lack of activation-coupled turnover [33,36–38]. Using HEK293E lysates, seven (#1, #3, #4, #5, #11, #12, #13) out of the 15 antibodies showed a specific band of the expected molecular weight in WT that was absent in *PRKN* KO cells (Figure 1). These antibodies also consistently gave a stronger PRKN signal in *PINK1* KO samples compared to WT further underscoring their specificity. The other eight antibodies either showed no signal or unspecific signal with no discernible difference between WT *PRKN* and KO samples.

**Figure 1.**
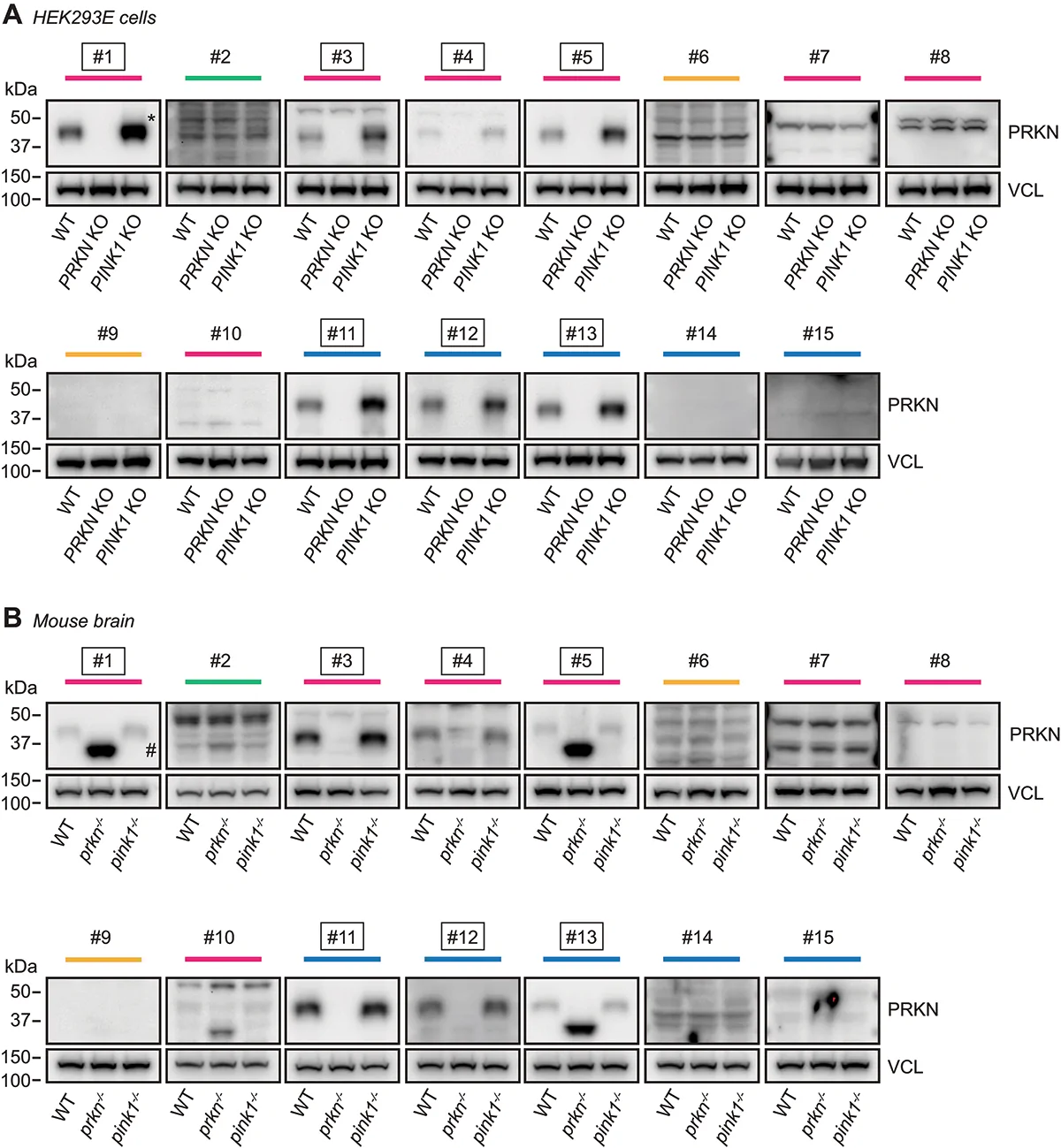
PRKN western blot assessment in HEK293E cell and mouse brain lysates. Western blots were performed using seven rabbit (pink), five mouse (blue), two goat (yellow) and one sheep (green) anti-PRKN antibodies in (A) HEK293E cells and (B) mouse brain lysates. VCL was used as a loading control. Full-length PRKN protein of the correct molecular weight is indicated with an asterisk in the top left image of (A). A lower molecular weight band that corresponds to the N-terminal stub of PRKN1-95 fused to EGFP in *prkn^−/−^* mice is marked with a hashtag in (B). Antibodies that successfully recognized PRKN are indicated with rectangles.

The same seven antibodies that gave a specific signal with HEK293E cell lysates also gave positive signal using mouse brain lysates. Bands slightly below the 50-kDa marker band were visible only for lysates from WT and *pink1*^−/−^ mouse brain but absent in the *prkn*^−/−^ samples. Consistent with their N-terminal epitope, some of the antibodies (#1, #5, and #13) further showed strong immunoreactivity against the N-terminal stub of PRKN (amino acids 1–95) that is fused to GFP in *prkn*^−/−^ animals [33,39]. Antibody #10 also showed reactivity to a band with a similar but slightly lower molecular weight. In summary, less than 50% of the tested antibodies were specific and sensitive enough to detect endogenous PRKN protein by western blot.

### PRKN sandwich ELISAs using recombinant protein

Different combinations of antibodies were next tested in a sandwich ELISA format using recombinant human full-length PRKN protein. To ensure that PRKN protein was accessible for antibody binding, we heat-denatured the recombinant protein prior to antigen-binding and then diluted to a range between 0–100 ng/ml (Figure 2). Seven rabbit, one sheep and two goat PRKN antibodies were used as capturing agents and combined with the five mouse PRKN antibodies as detecting agents. Antibodies #1, #3, #4 and #5, which showed a specific signal in the western blot analysis, also resulted in high, antigen concentration-dependent electrochemiluminescence (ECL) signal in the sandwich ELISA. In addition, antibody #2, a polyclonal sheep antibody which is not suitable for western blots according to the manufacturer, also gave high ECL levels in the sandwich setting. In contrast, antibodies #6–10 neither worked well for western blot nor for ELISA, regardless of the PRKN detecting antibody used. In general, the greatest signal in the sandwich setting was obtained with detecting antibody #11, followed by #12 and #13. However, when applied in combination with capture antibody #3, detection antibody #13 outperformed antibody #11. In contrast to detecting antibodies #11–13, the ECL signal obtained with antibodies #14 and #15 stayed close to the buffer blank even in presence of high concentration of recombinant PRKN protein (Figure 2).

**Figure 2.**
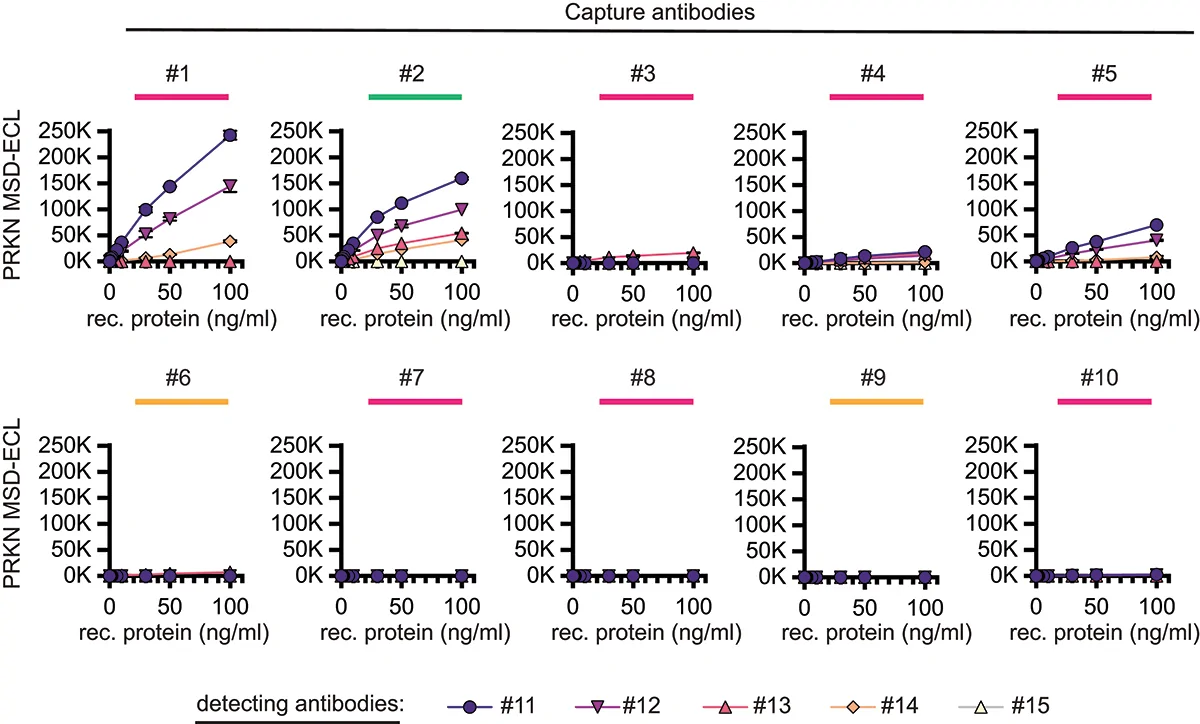
Assessment of PRKN antibody pairs for sandwich ELISAs using recombinant full-length human PRKN protein. Primary PRKN antibodies are numbered and color-coded by species according to Table 1. Antibodies from rabbit, sheep, and goat (#1–10) were used as capturing agents and each combined with mouse anti-PRKN detecting antibodies (#11–15) to detect different concentrations of recombinant full-length human PRKN protein: 100, 50, 30, 10, 6.0, 2.0, 1.2, 0.4, 0.24, 0.08, 0.016, or 0 ng/ml. Shown are uncorrected raw data as MSD-ECL from duplicate wells ± SD grouped by capture antibody. All tested antibody combinations had excellent coefficients of variations for duplicate wells (<5%) except for antibody #5 combined with antibody #14 (CV = 12.7%). MSD-ECL – Meso Scale Discovery-electrochemiluminescence; SD – standard deviation; CV – coefficient of variation.

Given the auto-inhibited conformation and major structural rearrangements of PRKN upon activation, we next compared heat-denatured versus non-heat-denatured samples for ELISA. We noted a 1.2 fold signal increase in presence of heat-denatured over non-heat-denatured PRKN or pS65-PRKN recombinant protein (Fig. S2A). These differences in MSD-ECL signal between heat-denatured and non-heat-denatured HEK293E cell lysates increased to ~ 2 fold in absence of PINK1 while values obtained with CCCP-treated WT cells were hardly affected by the heat-denaturation (Fig. S2B). Based on this outcome we decided to heat-denature samples from all sources for the PRKN ELISA.

### PRKN sandwich ELISAs using human cell and mouse brain lysates

To further validate our initial findings using human recombinant PRKN protein, we next tested the same antibody combinations using human cell (Figure 3A) or mouse brain lysates (Figure 3B). The best results with human cell lysates based on the fold change between WT and *PRKN* KO samples, were obtained with antibody pairs #1/#11, #2/#11 and #2/#12 all of which showed a fold change ≥ 5 (Figure 3A). This is generally in line with the results from the human recombinant protein, except that antibody pair #2/#11 outperformed antibody pair #1/11. Interestingly, with mouse brain samples, antibody pairs #3/#13 and #4/#11 showed the highest fold-change of 2.5 between WT and *prkn*^−/−^ samples (Figure 3B) while the antibody pairs that worked best with human cells resulted in lower fold changes between 1.7–2.0. Of note, the fold-changes that were received with human HEK293E cells were generally higher compared to mouse brain lysates except for antibody combination #3/#13, which showed a comparable outcome in both species. Antibody pair #4/#11 appeared to target mouse better than human PRKN based on MSD-ECL ratios in both species. On the other hand, antibody pairs #1/#11 and #2/#11 performed better with human compared to mouse samples, which suggests that these two antibody combinations preferentially recognize human over mouse PRKN protein.

**Figure 3.**
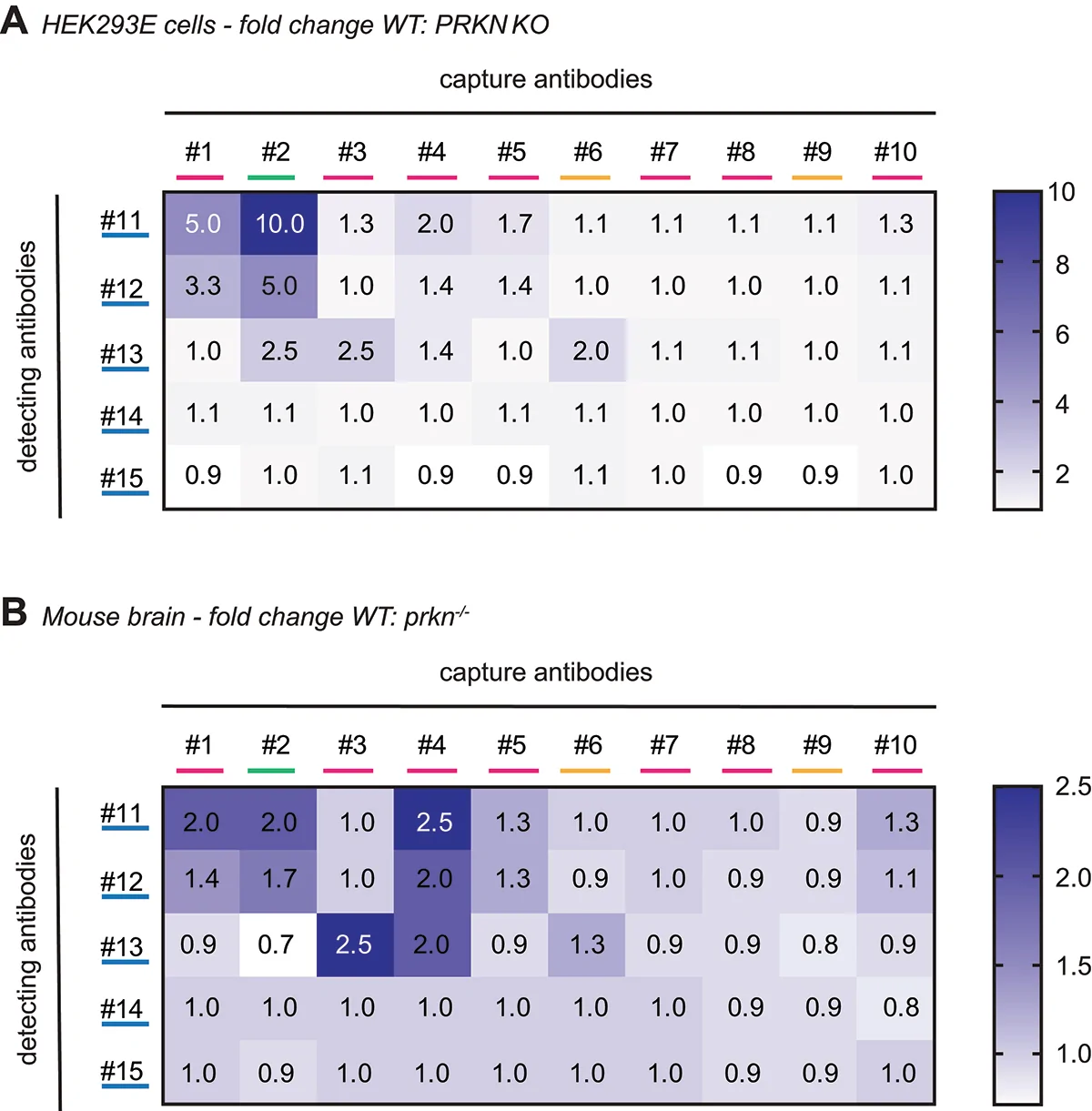
Assessment of PRKN antibody pairs using HEK293E cell and mouse brain lysates. Pairs of PRKN antibodies (rabbit, goat, and sheep for coating and mouse for detecting) were tested in a sandwich ELISA format using (A) HEK293E lysates from WT and *PRKN* KO cells or (B) mouse hemibrain lysates from WT and *prkn*^−/−^ animals. Shown are heatmaps of the Fold changes between WT and *prkn*^−/−^ samples. Primary PRKN antibodies are numbered and color-coded by species according to Table 1.

### Analytical sensitivity and linearity assessment of PRKN sandwich ELISAs

To test the most promising two antibody pairs for human detection (#1/#11 and #2/#11) and the best two for mouse detection (#3/#13, #4/#11) in further detail, we determined the Limit of Blank (LoB), the Limit of Detection (LoD), and the Limit of Quantification (LoQ) using human recombinant full-length PRKN protein (Table 2). The LoB, which represents the noise that is detected in the complete absence of PRKN protein in a matrix-dependent manner, was very low for all four antibody pairs (<130 MSD-ECL). The lowest analyte concentration that is likely distinguishable from the blank was also very similar for all four antibody pairs (LoD <144 MSD-ECL). The LoQ was determined as the lowest concentration of analyte that could be measured with a predefined goal for imprecision (%CV <5%). The LoQ was below 194 fM for antibody pairs #1/#11 and #2/#11, between 194 fM and 1 pM for antibody pair #3/#13 and below 100 pM for antibody pair #4/#11. The range of linearity ranged from 0–100 ng/ml for antibody pairs #1/#11, #3/#13, #4/#11 and from 0–50 ng/ml for antibody pair #2/#11. The coefficient of variation was excellent for all four antibody pairs and below 5% (Table 2).

**Table 2.** Analytical sensitivity and linearity assessment of PRKN ELISAs.

ELISA	Ab pairs	LoB	LoD	LoQ	Linearity	%CV
PRKN	#1/#11	128	143	<10 pg/ml (<194 fM)	0–100 ng	2.5
PRKN	#2/#11	106	108	<10 pg/ml (<194 fM)	0–50 ng	2.2
PRKN	#3/#13	105	108	10–50 pg/ml (194–968 fM)	0–100 ng	2.3
PRKN	#4/#11	122	124	<100 pg/ml (<1.9 pM)	0–100 ng	3.5
p-S65-PRKN	#A/#11	302	318	<10 pg/ml (<194 fM)	0–100 ng	2.9

Sensitivity, linearity and robustness were evaluated using data obtained with three independent repeats using recombinant protein. LoB – Limit of Blank, LoD – Limit of Detection, LoQ – Limit of Quantitation, CV – coefficient of variation.

We next tested the same antibody pairs using lysates from untreated WT compared to *PRKN* and *PINK1* KO HEK293E cells, respectively. All four antibody combinations were able to differentiate between all genotypes in a significant manner although #4/#11 showed a lower significance level between WT and *PRKN* KO cells compared to the other antibody pairs (Figure 4A). When comparing overall fold changes between the genotypes, antibody pair #2/#11 performed best followed by #1/#11 (Table 3). The same antibody pairs were then tested using hemibrain lysates of WT, *Prkn*^+/-^ and *prkn*^−/−^ mice (Figure 4B). All antibody combinations resulted in statistically significant differences between all genotypes and thus were able to distinguish between one and two copies of the *prkn* gene. However, compared to antibody pairs #1/#11 and #2/#11, antibody pairs #3/#13 and #4/#11 resulted in a better separation between the genotype groups (Table 3).

**Figure 4.**
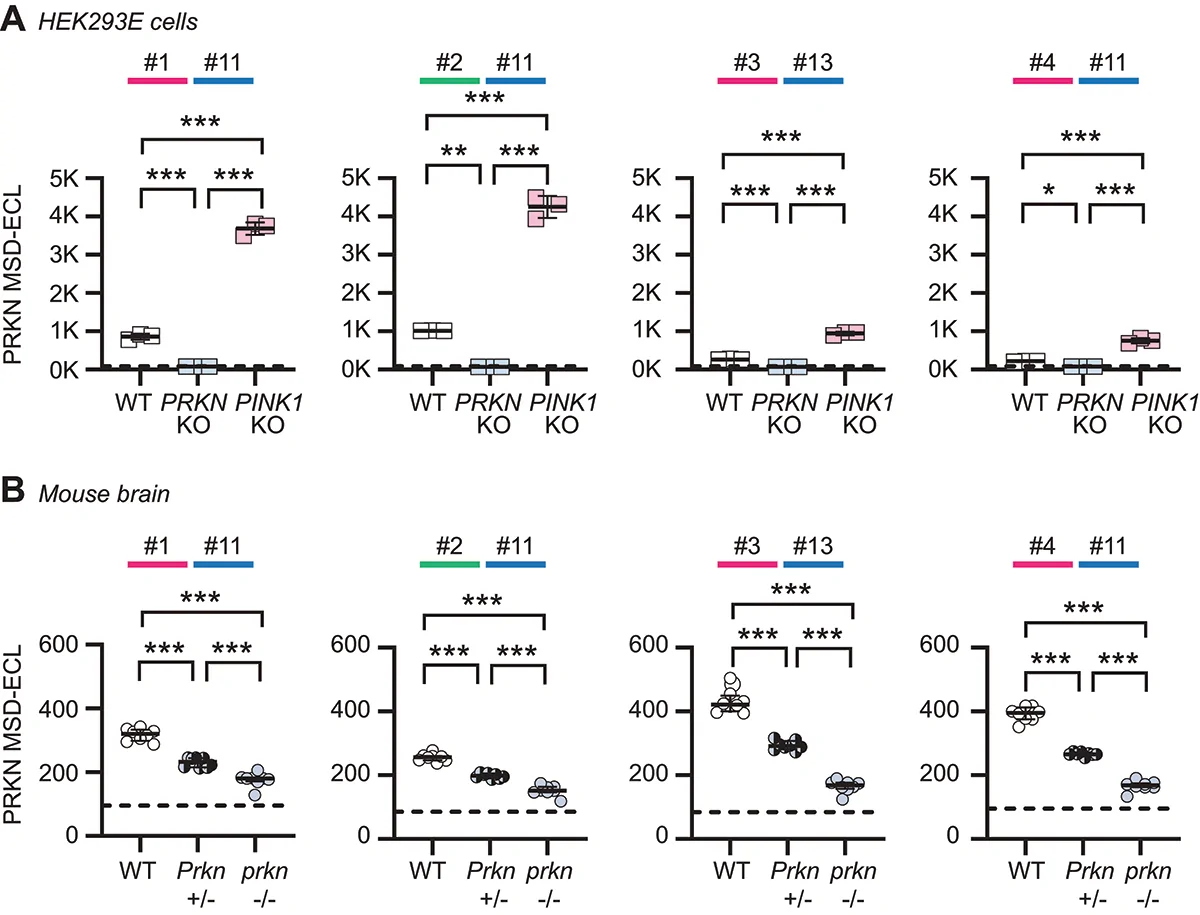
Detailed evaluation of four PRKN antibody pairs in cell and mouse hemibrain lysates. The four antibody pairs that gave the highest fold changes for human or mouse samples (#1/#11; #2/#11; #3/#13; #4/#11) were tested using (A) WT, *PRKN* KO and *PINK1* KO HEK293E cell lysates (*N* = 3 experiments); (B) 3-months-old WT, *Prkn^+/-^* and *prkn^−/−^* mouse hemibrain lysates (*N* = 8 per genotype). Shown are uncorrected mean raw data as MSD-ECL from duplicate wells ± SD. Dashed line shows the MSD-ECL signal of the buffer blank. Statistical analysis was performed using one-way ANOVA plus Tukey’s post-hoc test (***p <0.001; **p <0.005; *p <0.05). MSD-ECL – Meso Scale Discovery-electrochemiluminescence; SD – standard deviation.

**Table 3.** Comparison of antibody pairs using human and mouse samples.

		antibody pairs			
Biospecimen	Genotype comparison	#1/#11	#2/#11	#3/#13	#4/#11
HEK293E	WT vs. *PRKN* KO	10.5	**14.3**	3.9	2.8
	*PINK1* KO vs. WT	**4.3**	4.2	3.6	3.5
	*PINK1* KO vs. *PRKN* KO	44.9	**59.9**	13.7	9.6
Mouse brain	WT vs. *Prkn*^*+*/-^	1.4	1.3	**1.5**	**1.5**
	WT vs. *prkn*^−/−^	1.8	1.7	**2.6**	2.4
	*Prkn*^*+*/-^ vs. *prkn*^−/−^	1.3	1.3	**1.8**	1.6

Antibody combinations were used to assess differences in total PRKN levels between different genotypes of human and mouse samples. The average of three independent experiments was used for HEK293E cells. For mouse brain, *N* = 8 samples from 3-months-old animals were used per genotype. Shown is the fold change between the indicated genotypes.

In conclusion, antibody pairs #1/#11, #2/#11 and #3/13, #4/#11 are the best options for human and mouse samples, respectively. Since both antibodies in pairs #1/#11 and #4/#11 are monoclonal, these pairs may offer greater consistency in commercial availability over #2/11 and #3/13.

### p-S65-PRKN sandwich ELISA

To develop an ELISA specifically for activated PRKN, we used several phospho-specific p-S65-PRKN capture antibodies from rabbit and paired them with the best mouse detection antibodies from the total PRKN ELISA (Table 1). Of the five p-S65-PRKN antibodies tested, only antibody #A gave a strong signal when using heat-denatured, recombinant p-S65-PRKN protein as the antigen (Figure 5A). While antibody #A showed a very strong preference for p-S65-PRKN over PRKN, at very high tested concentrations (>30 ng/ml) there was a small increase of signal also with non-phosphorylated PRKN for both #A/#11 and #A/#13 about 1.5–1.8-fold over background levels. We further tested antibody #A using a direct ELISA approach and confirmed the specificity for p-S65-PRKN as no signal was obtained with p-S65-Ub in either monomeric or chain-linked form (Fig. S3A). We also confirmed that heat-denatured samples give a greater signal compared to non-heat-denatured samples (Fig. S3B) and used heat-denatured samples for all subsequent experiments.

**Figure 5.**
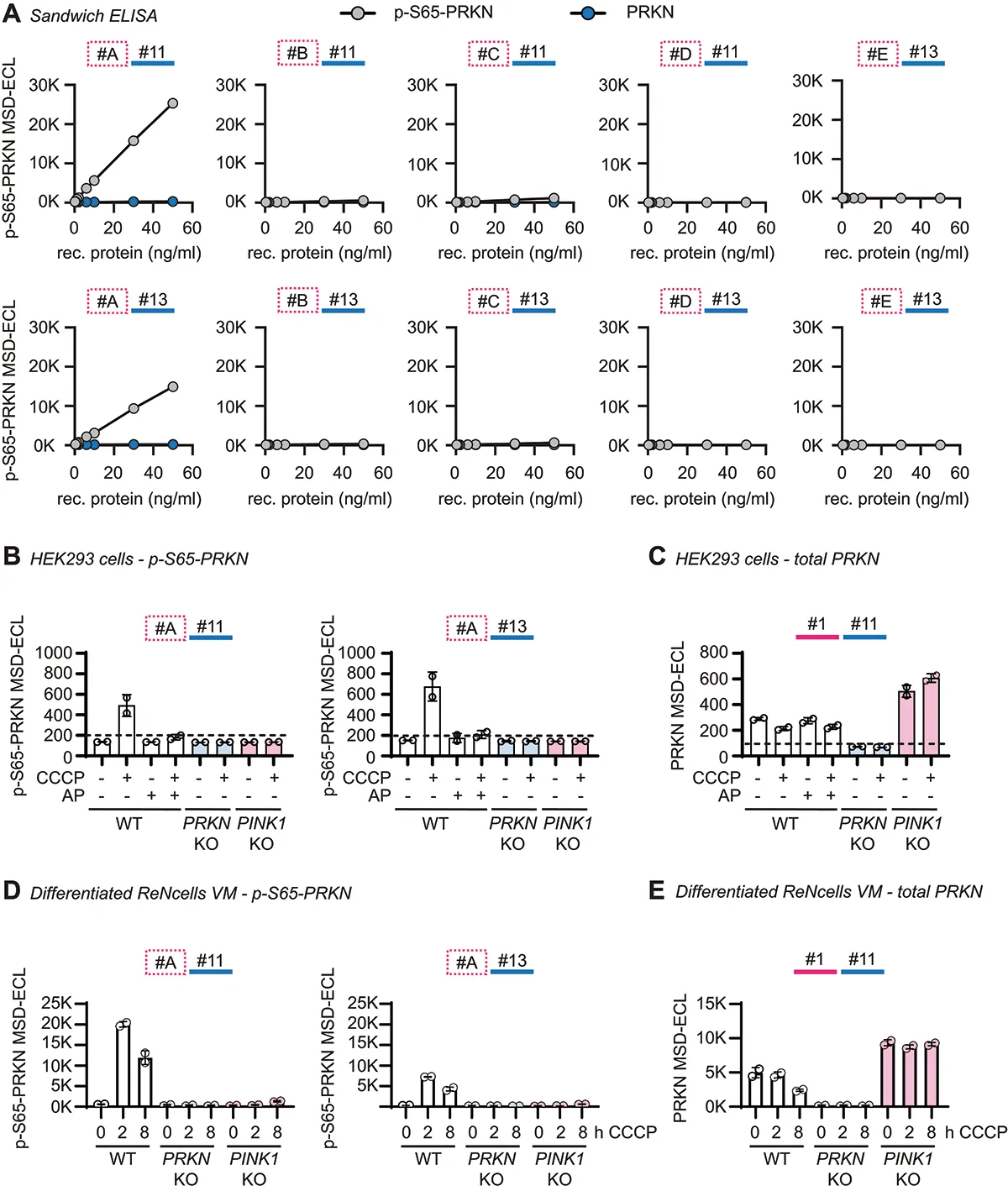
p-S65-PRKN ELISA assessment using recombinant protein, HEK293E cells and differentiated ReNcell VM. (A) five rabbit p-S65-PRKN antibodies (#A-E) were used as capturing agents and combined with the best performing two mouse detecting antibodies (top row #11; bottom row #13) using phosphorylated (p-S65; gray) and non-phosphorylated (S65; blue) recombinant full-length human PRKN protein in different concentrations: 50, 30, 10, 6.0, 2.0, 1.2, 0.4, 0.24, 0.08, 0.016, or 0 ng/ml. Shown are the mean raw MSD-ECL values from duplicate wells per measurement. (B) the two promising p-S65-PRKN ELISA antibody combinations (#A/#11; #A/#13) were further validated in HEK293E cells. WT, *PRKN* KO and *PINK1* KO HEK293E cells were treated for 0 or 5 h with CCCP and split lysates either dephosphorylated for 2 h, 37°C with alkaline phosphatase (AP+) or kept on ice with phosphatase inhibitors (AP-). (C) total PRKN ELISA (#1/#11) served as negative control and was not impacted by the enzymatic treatment. Shown are the mean ± SD from two independent experiments (B,C). (D) the same antibody pairs as in (B) were validated using lysates of differentiated ReNcells VM WT, *PRKN* KO or *PINK1* KO that were treated for 0, 2 and 8 h with the mitochondrial depolarizer. (E) total PRKN ELISA (#1/#11) served again as negative control with PRKN showing a different distribution across genotypes from both p-S65-PRKN ELISAs. Shown are the mean ± SD from two independent experiments (D,E). Dashed line in B-E indicates levels of buffer blank.

To further validate the phospho-specificity of the p-S65-PRKN ELISA, we tested both p-S65-PRKN ELISAs (#A/#11; #A/#13) on HEK293E cell lysates. WT, *PRKN* KO and *PINK1* KO HEK293E cells were treated with or without the mitochondrial depolarizer CCCP for 5 h to induce phosphorylation of PRKN. CCCP treatment of PRKN WT cells led to a ~ 4 times greater signal with both p-S65-PRKN antibody pairs #A/#11 and #A/#13 (Figure 5B). Importantly the signal increase was absent in samples that had been dephosphorylated by addition of alkaline phosphatase, and in lysates obtained from *PRKN* or *PINK1* KO cells, providing evidence that both antibody pairs specifically recognize S65-phosphorylated PRKN in cells. As expected, the total PRKN signal as assessed by the antibody pair #1/#11 was unaffected by dephosphorylation and was absent in *PRKN* but not in *PINK1* KO cells (Figure 5C).

Using neurons that were differentiated from the progenitor line ReNcell VM, we also observed a signal increase for p-S65-PRKN with CCCP treatment (Figure 5D). Interestingly, the p-S65-PRKN signal was greater 2 h after CCCP treatment compared to 8 h CCCP, indicating that p-S65-PRKN levels might not plateau but rather peak and then decrease. Consistent with HEK293E cells and further validating the specificity of the assay, there was no signal in lysates obtained from neurons without PINK1 or PRKN. Compared to HEK293E cells, MSD-ECL values were higher in differentiated ReNcell VM, consistent with greater total PRKN levels in the neurons (Figure 5E).

We next confirmed a coefficient of variation of below 5%, linearity between 0 and 100 ng as well as a LoQ of <10 pg/ml using human recombinant pS65-PRKN protein (Table 2). Together, this confirms that the identified antibody combination of antibody #A/#11 results in a pS65-PRKN specific immunoassay with excellent analytical characteristics.

### Acute mitochondrial depolarization leads to ~50% PRKN phosphorylation in neurons

To determine how much of PRKN protein is phosphorylated in CCCP-treated neurons at any given time, we generated absolute values for PRKN and p-S65-PRKN from a 12-point standard curve prepared with recombinant p-S65-PRKN protein. While different matrices used to dilute the standards did not change the outcomes for the p-S65-PRKN assay, the use of PRKN KO cell lysates lowered concentration outcomes compared to blocking buffer for the total PRKN assay (Fig. S4). Since this may be particularly relevant for absolute quantifications of p-S65-PRKN versus total PRKN in cell culture samples, we decided to use PRKN KO cell lysates as the more similar matrix for the standard curves.

We determined the absolute levels of total and p-S65-PRKN from differentiated PRKN WT neurons that were treated with CCCP in a time-course experiment (Figure 6A). Total PRKN levels in differentiated ReN VM neurons started at 17.0 ng/mg total protein at baseline, 8 to 11 times higher compared to human skin fibroblasts and HEK293E cells, respectively (Table 4). Upon CCCP treatment of neurons, total PRKN levels increased slightly within the first 30 min to 18.8 ng/mg total protein and declined continuously afterward with CCCP-treatment to 2.2 ng/mg total protein after 24 h consistent with PINK1-activity dependent turnover of PRKN. Levels of p-S65-PRKN increased maximally with CCCP-treatment from basically 0 to 8.5 ng/mg total protein after 2 h of CCCP treatment, and then continuously declined to 1.4 ng/mg total protein after 24 h CCCP-treatment. Interestingly, after the initial induction to 42% after the first hour of CCCP treatment, the ratio of p-S65-PRKN to total PRKN stayed at about 50% over the entire time course (Figure 6B).

**Figure 6.**
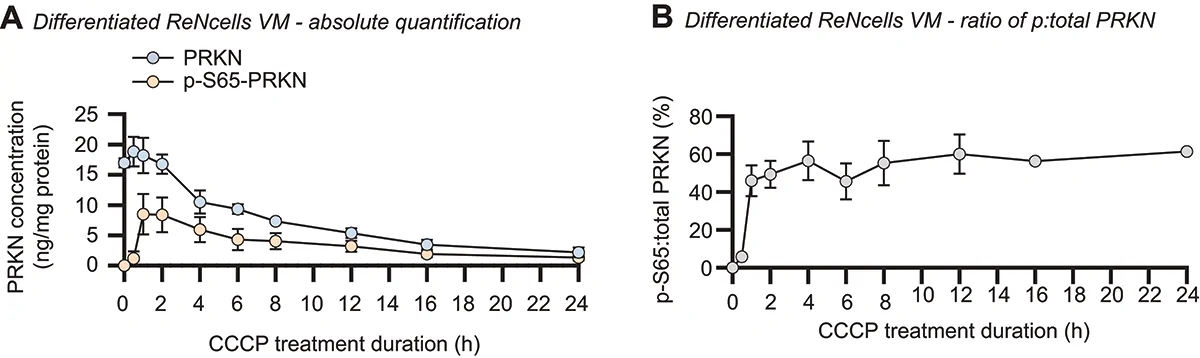
Absolute concentrations of p-S65-/total PRKN in differentiated ReNcell VM. (A) absolute p-S65-/total PRKN quantification in CCCP-treated differentiated ReNcells VM over time using antibody pairs #1/#11 for total PRKN ELISA and #A/#11 for p-S65-PRKN. Differentiated ReNcells VM were treated for the indicated times with CCCP and 10 µg of total protein was loaded per well. Recombinant p-S65-PRKN protein diluted in *PRKN* KO cell lysate was used to generate a standard curve for both total- and p-S65-PRKN ELISA. Shown is the mean ± SD from three independent experiments. (B) the fraction of p-S65-PRKN as a percentage of total PRKN in differentiated ReNcells VM is shown over time with CCCP-treatment and based on the three biological replicates shown in (A). MSD-ECL – Meso Scale Discovery-electrochemiluminescence; SD – standard deviation.

**Table 4.** Absolute PRKN concentrations in cells.

Cell line	PRKN concentration (ng/mg total protein)	Std.Dev
HEK293E	1.59	0.11
human fibroblasts	2.00	0.43
ReN VM neurons	16.98	0.73

PRKN concentrations were established using three independent experiments.

### Novel MSD ELISA suggest lower levels of PRKN in human brain with rare and common PRKN mutations

We finally applied the total PRKN ELISA to detect PRKN protein levels in samples from mutation carriers. We had previously shown by western blot analysis that the R275W mutation, a disease-associated rare missense mutation in *PRKN*, leads to a considerable reduction of PRKN protein [40]. Here, we confirmed an ~80% reduction of PRKN protein in gene-edited ReNcells VM with biallelic PRKN^R275W^ mutation compared to isogenic WT cells using antibodies #1 and #11 for the PRKN ELISA (Figure 7A). To exclude the possibility that the observed reduction reflects altered antibody recognition rather than true differences in protein abundance, we performed additional western blot analyses using two different antibodies (#3 and #13) targeting distinct epitopes (Figure 7B). These experiments demonstrated markedly reduced PRKN levels in PRKN^R275W^ cells, supporting that the decrease reflects reduced protein abundance rather than epitope-specific detection bias. In both the ELISA and western blot experiments, we observed PINK1-activity dependent degradation of PRKN over time treatment (Figure 7A,B). In line with our published report, we also detected lower total and absent p-S65-PRKN in skin fibroblasts from PD patients with PRKN^R275W/Q^ mutation (Fig. S5). We next compared PRKN protein levels in human midfrontal cortex samples from heterozygous carriers of the PRKN^R275W^ mutation compared to non-carriers with similar sex, age, and pathological characteristics. MSD-ECL levels were about ~100 times lower in the Triton X-100-soluble compared to the -insoluble (SDS) fraction (Figure 7B). Samples from PRKN^R275W^ carriers showed largely reduced protein levels in the insoluble fraction while there was a trend toward reduction also in the soluble fraction.

**Figure 7.**
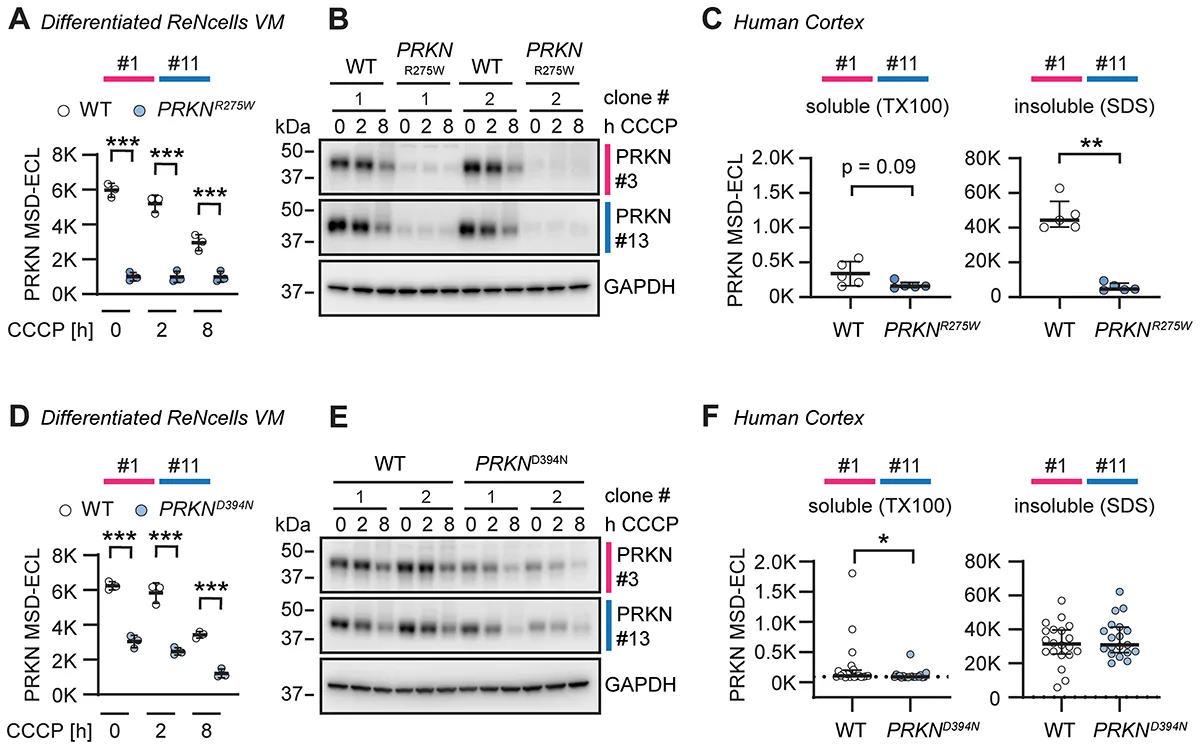
PRKN ELISA assessment in differentiated ReNcells VM and in human brain with rare and common *PRKN* mutations. All PRKN ELISAs were performed using antibody #1 (capture) and antibody #11 (detection). (A) total PRKN ELISA using lysates from differentiated ReNcells VM expressing WT PRKN or PRKN^R275W^ and treated for 0, 2, and 8 h with CCCP. Ten µg of total protein was loaded per well. Shown is the mean ± SD obtained with three biological cell clones. (B) representative western blot of two independent clones per genotype (WT and PRKN^R275W^) treated as in (A), probed with antibodies #3 and #13. VCL served as loading control. (C) total PRKN ELISA using lysates from human cortex samples of heterozygous PRKN^R275W^ carriers and non-carrier controls (*N* = 5 per group), matched for LBD/AD pathology, Braak tangle stage, Thal phase, sex, and age. Thirty µg of total protein was loaded per well. Soluble (TX100) and insoluble (SDS) fractions were analyzed using identical volumes. Data represent median ± interquartile range. (D) total PRKN ELISA using lysates from differentiated ReNcells VM expressing WT PRKN or PRKN^D394N^ and treated for 0, 2, and 8 h with CCCP were assessed in three independent experiments. Ten µg of total protein was loaded per well. Shown is the mean ± SD from three repeat experiments with two clones per genotype. (E) representative western blot of two independent clones per genotype (WT and PRKN^D394N^) treated as in (D), probed with antibodies #3 and #13. VCL served as loading control. (F) total PRKN ELISA using lysates from human cortex samples of heterozygous PRKN^D394N^ carriers and non-carriers (*N* = 20 per group). Thirty µg of total protein was loaded per well for the soluble (TX100) fraction; identical volumes were used for consecutive fractions. Data represent median with interquartile range. Statistical analysis for A, D was performed using two-way ANOVA plus Tukey’s test for multiple comparison (**p <0.005, ***p <0.001) and C, F using non-parametric mann-whitney test (*p < 0.05, **p < 0.01). MSD-ECL – Meso Scale Discovery-electrochemiluminescence; SD – standard deviation; sol. – soluble fraction (TX100); insoluble – insoluble fraction (SDS); CON – control.

In order to study if a common *PRKN* variant would also lead to a measurable effect, we introduced PRKN^D394N^ into ReNcell VM precursor cells using CRISPR-Cas9 and measured total PRKN levels. While not as pronounced as for PRKN^R275W^, there still was a robust and significant ~45% reduction of total PRKN levels in homozygous PRKN^D394N^ cells compared to WT (Figure 7C). This decrease was also observed in western blot analysis of two independent cell clones per genotypes using different antibodies (Figure 7E), suggesting that the observed effect is antibody binding independent. Over time CCCP treatment there was a reduction of PRKN protein levels, both in the WT and in the mutant cells (Figure 7D, E). Using available cingulate cortex samples from heterozygous PRKN^D394N^ carriers compared to age, sex, and pathology matched controls, we found significantly reduced PRKN levels, indicating that the D394N variant might change the availability of PRKN also in human brain (Figure 7F). While the change here was most pronounced in the soluble fraction, overall, the data indicates that both rare as well as common *PRKN* mutations may affect the levels of the PRKN protein, and this could have implications for PD susceptibility for carriers of these *PRKN* variants.

## DISCUSSION

The PINK1-PRKN mitophagy pathway has been recognized as a protective quality control mechanism with large therapeutic potential. First small molecule modulators are approaching clinical trials, but pathway-related disease and treatment biomarkers are still scarce [41]. Here we developed a novel set of sandwich ELISA methods to detect both total protein levels of the E3 Ub ligase PRKN and the PINK1-phosphorylated, activated form p-S65-PRKN. Our work establishes the first knockout-validated assays with reliable selectivity for the detection of endogenous mouse and human PRKN with a limit of quantification in the femtomolar range. Application of the new ELISAs revealed a relatively large effect size of a common genetic PRKN variant, strengthening the notion that the clinical significance of PRKN is likely underestimated. These findings indicate that, in addition to assessing genetic PRKN risk, its protein levels and activity must be biochemically determined and taken into consideration. Together with the recently established MSD ELISAs assays for PINK1 and their joint product p-S65-Ub [42,43], this will lead to an improved diagnosis, prognosis and patient stratification. Comprehensive evaluation of the PINK1-PRKN pathway under endogenous conditions in experimental models and in human biospecimens will better define basal and stress-induced activation and flux/degradation rates.

It is well established that bi-allelic loss-of-function mutations in *PRKN* are the most common genetic cause for early-onset PD [44]. However, the role of heterozygous *PRKN* mutations for disease risk on the population level remains a matter of debate [13,14]. Functional imaging of heterozygous *PRKN* mutation carriers is consistent with a mild dopaminergic nigrostriatal dysfunction, suggesting that a 50% loss of PRKN function might predispose individuals to PD [45]. The presence of copy number variants in *PRKN* is easy to miss with conventional short read sequencing, suggesting that the number of individuals with reduced PRKN expression is likely underestimated [46]. In many cell and animal models, PRKN broadly protects against various proteotoxic insults and aging phenotypes [47]. Moreover, PRKN acts as a tumor suppressor gene in several types of cancer where its loss of function or reduced expression is associated with tumor development and progression [48,49]. Thus generally, more PRKN is better, however interpreting PRKN by its total protein levels alone can be misleading. This is because autoinhibited PRKN is extremely stable and long-lived but rapidly turned over upon activation by PINK1 [32,34,50]. It is thus important to differentiate between total versus activated p-S65-PRKN.

Numerous cellular studies have demonstrated that the PINK1-PRKN pathway leads to the broad degradation of mitochondria in response to acute and complete membrane depolarization. However, it has become clear that in absence of such maximal activation, PINK1-PRKN signaling exhibits a much more subdued profile [33,51]. The low basal pathway activity can be appreciated by a subtle, but consistent accumulation of inactive PRKN protein in absence of the Ub kinase PINK1 in cells and animals [33]. While the specific fold increase in PRKN protein levels seems to depend on the relative gene expression and activation of both enzymes, PINK1 and PRKN, it also reflects the basal mitophagy rates of that cell or tissue type in the absence of stress. Due to this transient and highly dynamic nature, the measurement and analysis of PINK1-PRKN mitophagy is both technically challenging and complicated to interpret especially *in vivo*. However, measuring PINK1, PRKN, and p-S65-Ub together may allow the approximation of mitophagy even when temporal resolution is not available, for example, in human disease samples.

In order to develop an MSD ELISA against total PRKN, we screened 15 commercially available antibodies. Importantly, less than half of the tested antibodies recognized PRKN in western blot or MSD. This is in agreement with a report from Ycharos Inc, an open science, public interest company, stating that only about ~40% of tested PRKN antibodies are specific [52]. Seven antibodies (#1, #5, #7, #12, #13, #15, #16) were included in both studies and gave the same results, further providing confidence in the outcome. Remarkably, a current lot of the most-cited PRKN antibody according to the citeab (www.citeab.com) and antibodypedia (www.antibodypedia.com) websites did not recognize PRKN in either study. This further highlights the critical need to carefully evaluate antibodies and antibody-based tools, such as ELISAs, and to consider lot-to-lot variations especially when relying on polyclonal antibodies. In this regard it is worth mentioning that different formulations of the same antibody clone from independent manufacturers also showed differences, at least in some cases (Fig. S6).

Among the best four antibody pairs for the detection of total PRKN, two antibody pairs performed particularly well with human samples (#1/#11 and #2/#11), while the two other pairs worked better with mouse samples (#3/13 and #4/#11). Although none of the antibodies specifically recognized only human or mouse PRKN protein, which share 91% sequence identity, differences in affinity may be caused by small structural changes due to some variance in the primary amino acid sequence. However, we also cannot exclude the presence of different post-translational modifications in human or mouse PRKN, which may influence antibody affinity by masking epitopes or by creating ultra-affine neo-epitopes. Alternatively, it is possible that human and mouse samples have different matrix effects that impact antibody performance. It would be possible to test this using recombinant human vs mouse PRKN protein side-by-side in the future. Regardless, best performing antibody combinations targeted both PRKN’s C and N terminus (epitope for #4 is undetermined) and suggest steric hindrance of antibodies with similar epitope region in a sandwich approach.

In addition to a sensitive total PRKN ELISA, we further developed a first-in-class S65-phospho-specific PRKN sandwich ELISA that offers robust and reliable detection of activated PRKN. We tested five p-S65-PRKN antibodies in combination with the two best total PRKN detection antibodies and identified one phospho-specific antibody that worked particularly well for ELISA. The combination of antibody #A in combination with #11 resulted in a LoQ of <194fM with excellent CV. The specificity of this signal was further confirmed using PINK1 or PRKN KO cells as well as alkaline phosphatase treatments. It was remarkable that for both the total and the phospho-specific PRKN assay, there was a better discrimination between different treatment and genotype conditions using heat-denatured samples compared to non-heat-denatured samples, likely reflecting differences in epitope accessibility. Along the same line, levels of total PRKN protein seemed to slightly but significantly increase after just 30–60 min of CCCP treatment, the same time point where a strong increase of p-S65-PRKN occurred, suggesting that this spike could reflect the activation of PRKN as it is opening up from a “closed” inactive conformation [25,26]. Notably, the effect was observed in heat-denatured lysates, indicating that enhanced detectability may reflect activation-associated molecular changes that persist following denaturation. These might be caused by post-translational modifications or interaction states that transiently alter PRKN detectability in the assay.

The combined p-S65-PRKN and total PRKN ELISA offer new mechanistic insights of PRKN activation and PRKN turnover rates especially when used in time course settings that allow absolute quantification of both analytes. Here, we show that upon initial activation of PRKN, differentiated ReNcell VM maintain a steady ratio of ~50% p-S65-PRKN compared to total PRKN despite substantial PRKN degradation. Given our recent findings that the activation of PRKN likely leads to its consumption [34], similar to other self-regulating E3 Ub ligases [35], it is likely that p-S65-PRKN is short lived. Yet, while the activated form is rapidly eliminated, PINK1 continues to recruit and phosphorylate additional PRKN molecules which likely results in the observed balance of phosphorylated to non-phosphorylated PRKN when the pathway is maximally activated. The swift degradation of p-S65-PRKN might also explain why we were unable to detect significant amounts of p-S65-PRKN in brain under basal conditions when the pathway is mostly inactive.

Our study also establishes the quantitative assessment of PRKN in human brain tissues. It has been suggested that multiple post-translational modifications of PRKN such as nitrosylation, oxidation, and dopamine adducts render it inactive (reviewed in [18–22,53]) and may result in insolubility of the E3 Ub ligase in human brain [23,24]. In agreement with this, we found PRKN protein mostly in the Triton X-100 [TX100] insoluble (SDS soluble) fraction in human cortex. Previously we have established that the pathogenic missense mutation *PRKN*^R275W^ leads to a dramatic reduction in protein levels [40]. Here we validated these findings using the new PRKN ELISA and further confirmed the effect in human postmortem brain samples from heterozygous carriers of PRKN^R275W^.

Strikingly, we also detected an unexpected large effect size of a common PRKN variant present in ~4% of mostly Caucasians with European descent. PRKN protein levels were lower in both gene-edited neurons and in human autopsy brain samples from carriers of the PRKN^D394N^ variant with cells carrying a homozygous PRKN^D394N^ variant showing stronger and more consistent effect compared to heterozygous PRKN^D394N^ brain samples. Despite a CADD score of 22.9, this variant is currently defined as benign or likely benign although earlier literature already suggested a trend toward association with PD [54]. Together with recently identified functional deficits of a common PINK1 variant [55], the current results further emphasize the critical need to biochemically evaluate even supposedly benign variation in addition to purely genetic risk assignment.

As with any antibody-based method, epitope recognition may theoretically be influenced by post-translational modifications or amino acid substitutions within or near the targeted epitope. Such changes could alter antibody affinity by masking epitopes or generating neo-epitopes. Therefore, we cannot formally exclude that specific post-translational modifications or rare sequence variants may affect antibody recognition under certain pathological conditions and may require confirmation by orthogonal approaches such as mass spectrometry-based quantification. Nevertheless, we established the use of several different PRKN antibody pairs for ELISA, rigorously validated the assays using knockout controls and demonstrated consistent performance across multiple biological systems, supporting their reliability and robustness for quantitative assessment of endogenous PRKN. Importantly, the modular nature of the sandwich ELISA format also offers opportunities for future adaptation. For example, combining PRKN-directed capture antibodies with ubiquitin-specific detection antibodies (or vice versa) could, in principle, enable selective quantification of PRKN-ubiquitin conjugates. Such modifications may allow more direct assessment of PRKN ubiquitination or substrate engagement.

In conclusion, the MSD assays developed herein facilitate sensitive and reliable quantification of PRKN protein and enable determination of activation and turnover rates under endogenous conditions at baseline and upon stress. The novel ELISAs can be readily implemented alongside PINK1 and p-S65-Ub to enable broader investigation of the pathway across experimental models and human biospecimens. Initial applications suggest that the clinical significance of PRKN may be underestimated and that both genetic variation and protein abundance substantially influence disease risk and resilience in the wider population. Biochemical determination of clinically and therapeutically relevant PRKN levels, and understanding how activation status modifies risk, onset, and progression, will therefore be critical. We anticipate that these assays will contribute to improved diagnosis, prognosis, and patient stratification in the future.

## Material and methods

### Antibodies

The 15 total PRKN and five p-S65-PRKN antibodies used for ELISA development are listed in Table 1. We also compared different formulations of PRK8 (Cell Signaling Technology, 4211; Biolegend, 808501; Santa Cruz Biotechnology, sc-32282) to antibody #11 (Millipore, MAB5512) used for ELISA development and for clone 21H24L9 (Invitrogen, 702785) we also tested an engineered version (Invitrogen, 740019 R).

### Recombinant proteins

Recombinant total PRKN (UB317) and p-S65-PRKN (UB317A) proteins were purchased from Life Sensor. Recombinant Ub monomer (U100H), p-S65-Ub monomer (U-102), K48 p-S65-Ub_4_ (UC-250), K63 p-S65-Ub_4_ (UC-350) were purchased from Boston Biochemicals. Upon arrival, recombinant proteins were dissolved, aliquoted and stored at −80°C. On the day of use, aliquoted proteins were diluted to a concentration of 1 ng/µl in RIPA buffer (50 mM Tris, pH 8.0 [Affymetrix, 75825], 150 mM NaCl [Fisher Scientific, BP-358], 0.1% SDS [Bio-Rad, 1610302], 0.5% deoxycholate [Sigma-Aldrich, D6750], 1% NP-40/Igepal [United States Biochemical, 19628] supplemented with 2% SDS and fully heat-denatured by incubating for 5 min at 95°C. Denatured proteins were diluted 10-fold in blocking buffer (1% nonfat milk (Sysco, 5398953) in TBS-T (TBS [Boston BioProducts, BM301] plus 0.1% Tween 20 [Sigma, P1379]) to a final concentration of 0.2% SDS.

### Cell cultures and treatment

Human embryonic kidney cells 293E (Hek293E [ATCC, CRL-3216]) were cultured in Dulbecco’s modified Eagle medium (Gibco, 11965118) supplemented with 10% FBS (Neuromics, FBS001800112). *PINK1* and *PRKN* KO HEK293E cells have been described previously [43].

ReNcell VM human neuronal progenitors (Millipore, SCC008) were cultured on Matrigel (Corning, 354230)-coated dishes in DMEM/F12 (Gibco, 10565042) containing B27 (Gibco, 17504044), 5 U/ml heparin (Sigma Aldrich, H3149), and 50 µg/ml gentamicin (Gibco, 15750060) in the presence of 20 µg/ml EGF (epidermal growth factor; Peprotech, AF-100–15) and FGF (fibroblast growth factor; Peprotech, 100–18B). *PRKN* and *PINK1* KO ReNcell VM were described previously [33,34]. Additional gene-editing of ReNcell VM was performed as described below. Differentiation of ReNcell VM into neurons was performed by withdrawal of growth factors in the presence of 2 ng/ml GDNF (Peprotech, 450–10) and 1 mM cAMP (Invivochem, V1846) for 15 days.

Primary human dermal fibroblasts from individuals with *PRKN*^R275W/R275Q^ or homozygous *PINK1*^Q456X^ have been described previously [40,56]. Fibroblasts from a healthy relative of the PINK1^Q456X^ mutation carrier were used as controls [56]. Fibroblasts were cultured in Dulbecco’s modified Eagle medium supplemented with 10% fetal bovine serum, 1% PenStrep (Gibco, 15140122), and 1% non-essential amino acids (Gibco, 11140050).

All cells were grown at 37°C, 5% CO_2_:air in humidified atmosphere. To induce mitochondrial depolarization cells were treated with 20 µM CCCP (Sigma, C2759) or 1 µM valinomycin (Cayman Chemical, 10009152). The analysis of fibroblasts was conducted in accordance with and approved by the Institutional Review Board of Mayo Clinic (20–011781, approved 01/6/2021).

### Genome-editing with CRISPR-Cas9

ReNcell VM carrying a homozygous PRKN^D394N^ mutation were generated by CRISPR-Cas9 meditated gene editing. The guideRNA duplex was hybridized *in vitro* from a custom crRNA with the target sequence (5’-ACAGAGTCGATGAAAGAGCC-3’) and a tracrRNA (Integrated DNA Technologies, 1072533) and mixed with recombinant Cas9 protein (Integrated DNA Technologies, 1081061) according to the manufacturer’s instructions. The complex and a 200 base single stranded oligodeoxynucleotide was delivered into the ReNcells VM using a P3 nucleofector solution (Lonza, V4XP-3032) and nucleofector X device (Lonza, Basel, Switzerland) using program settings CL148. For single clone selection and screening, cells were plated in 96-well plates by limited dilution. Media with growth factors was added every 2–3 days for the next 14 days. When cell clones became apparent, cells were detached with Accutase (Innovative Cell Technologies, AT104-500) and 20% of the cell suspension was re-plated. The remainder of the cell suspension was used for DNA extraction followed by PCR amplification of the target region and restriction enzyme digest to prescreen the clones. Successful targeting was confirmed by Sanger sequencing of both strands using the following primers: 5`-GAACATAAACTCTGATCCC-3,’ 5’- CATTAATCGAGGGGTTAC-3`.

### Cell lysis and western blotting

Cells were washed twice with ice-cold PBS and harvested in 1x RIPA buffer containing protease inhibitor cocktail and phosphatase inhibitors (Sigma-Aldrich, 11697498001 and 04906837001). Cell lysates were then cleared for 15 min, 4°C at 20,817 × g and protein concentrations were determined by a bicinchoninic acid assay (Thermo Scientific, 23225). For testing the specificity of the p-S65-PRKN ELISA, some cells were lysed in the absence of phosphatase inhibitors. These lysates were then split into two halves that were either treated with phosphatase inhibitors (Sigma-Aldrich, 04906837001) on ice or with alkaline phosphatase (Thermo Scientific, EF0651) for 2 h at 37°C and 700 rpm. All cell lysates were subsequently centrifuged for 10 min, 4°C at 20,817 × g and protein concentrations were determined by bicinchoninic acid assay. Thirty µg of lysate were diluted in Laemmli buffer (62.5 mM Tris, pH 6.8, 1.5% SDS, 8.33% glycerol [Fisher Scientific, BP2291], 1.5% β-mercaptoethanol [Sigma-Aldrich, M3148], 0.005% bromophenol blue [Sigma-Aldrich, B5525]), loaded on to 8–16% Tris-Glycine gels (Thermo Scientific, XP08165BOX) and transferred onto polyvinylidene fluoride membrane membranes (Millipore, IEVH00005). Membranes were then blocked for 1 h in 5% nonfat milk in TBS-T (TBS [Boston BioProducts, BM301] plus 0.1% Tween 20 [Sigma, P1379]) and incubated in primary antibodies overnight in 5% nonfat milk in TBS-T at 4°C. All antibodies were used in a concentration of 1 μg/ml except #3, which was used at a concentration of 200 ng/ml. VCL (vinculin) (Sigma-Aldrich, V9131; 0.016 µg/ml) was used as loading control. The following day, membranes were incubated for 1 h at room temperature with horse-radish peroxidase-coupled secondary antibodies: donkey anti-mouse IgG (Jackson Immunoresearch, 715–035-150), donkey anti-rabbit IgG (Jackson Immunoresearch, 711–035-152), goat anti-mouse IgG1 (Jackson Immunoresearch, 115–035-205), goat anti-mouse IgG2a (Jackson Immunoresearch, 115–035-206), and goat anti-mouse IgG2b (Jackson Immunoresearch, 115–035-207), rabbit anti-sheep IgG (Invitrogen, 31480), donkey anti-goat IgG (Santa Cruz, sc-2020). Proteins were visualized using Immobilon Western Chemiluminescent HRP Substrate (Millipore, WBKLS0500) on a Chemidoc MP imaging system (Bio-Rad, Hercules, CA, USA).

### Tissue collection and preparation of mouse and human brain lysates

A total of 24 C57BL/6 mice (8 WT (4 M/4F), 8 *Prkn*^+/-^ (4 M/4F), 8 *prkn*^*-/-*^ (4 M/4F)) three months of age were anesthetized and intracardially perfused with cold PBS (Invitrogen, 14190–235). Brains were removed and immediately flash-frozen in liquid nitrogen. Frozen brains were stored at −80°C and kept on dry ice and completely frozen during handling including weighing. All procedures involving animals were in accordance with the ethical standards established by Mayo Clinic and approved by the Institutional Animal Care and Use Committee at Mayo Clinic.

Frozen human frontal cortices from 5 cases (2 M/3F) with monoallelic PRKN^R275W^ mutation and frozen cortex samples (10 M/10F) with common PRKN^D394N^ variant were compared to cases with similar age at death, sex distribution and pathological characteristics without PRKN mutation (Table 5). All human cases were obtained from the Mayo Clinic Florida Brain Bank. Autopsies were performed after approval by the legal next-of-kin. Research on de-identified postmortem brain tissue is considered exempt from human subjects’ regulations by the Mayo Clinic Institutional Review Board.

**Table 5.** Subject characteristics of postmortem brain cohorts.

Cohort	N	Male (%)	Age median (min, max)	Braak tangle stage median (min, max)	Thal amyloid phase median (min, max)
**R275W cohort**					
control	5	2 (40%)	87 (71, 94)	V (III, VI)	5 (2, 5)
R275W	5	2 (40%)	94 (72, 99)	V (II, VI)	5 (0, 5)
**D394N cohort**					
control	20	10 (50%)	76 (59, 90)	IV (0, IV)	3 (0, 5)
D394N	20	10 (50%)	94 (72, 99)	III (0, IV)	3 (0, 5)

For protein extraction, frozen tissues were first homogenized in 5x volumes of ice-cold TBS buffer supplemented with protease and phosphatase inhibitors using glass Dounce tissue grinders (Fisher Scientific, K885300-0002). Tissue lysis was completed by adding ¼ volume of 5% TX100 to the TBS homogenates (final concentration 1%), pulse vortex for 30 s and 60 min incubation on ice. Brain lysates were subsequently cleared by two times centrifugation at 20,000 × g, 4°C for 15 min. The resulting supernatant was collected as TX100 soluble lysate and protein concentrations were determined by BCA assay. The TX100-insoluble pellet of human tissue was further washed twice with TX100, solubilized in 2% SDS containing TBS solution and then heated for 5 min at 95°C and subsequently centrifuged at 20,000 × g, 4°C for 15 min. The resulting supernatant was kept as SDS-soluble fraction. Volumes used for this fraction were identical to corresponding volumes for each TX100 fraction. Throughout all tissue lysis/homogenization steps, mouse and human brain tissues/lysates were kept at 4°C.

### PRKN and p-S65-PRKN sandwich MSD ELISA

For the PRKN MSD sandwich ELISA we used rabbit, goat and sheep total PRKN antibodies as capturing agents and combined them with mouse total PRKN antibodies as detecting agents. For the PRKN MSD sandwich ELISA we used rabbit p-S65-PRKN antibodies as capturing agents and combined them with mouse total PRKN antibodies as detecting agents. All capturing antibodies were used in a final concentration of 1 µg/ml (except antibody #3 in a final concentration of 200 ng/ml) in 200 mM sodium carbonate buffer (Sigma-Aldrich, S4132) pH 9.7 and coated overnight at 4°C with 30 µl per well in 96-well plates (Meso Scale Diagnostics, L15XA-3). The next morning plates were washed two times with TBS-T and then incubated with 300 µl blocking buffer (150 mM Tris, pH 7.4, 150 mM NaCl, 0.1% Tween-20, 1% nonfat milk) for 1 h at 22°C without agitation. Lysates from cells, mouse and human tissue were run as duplicates with 30 µg total protein per well unless specified otherwise. Lysates and recombinant proteins were fully denatured on a thermomixer at 95°C for 5 min in the presence of 2% SDS. Protein solutions were subsequently cooled down on ice and centrifuged for 2 min, 20,000 × g at 4°C before diluting them in blocking buffer. This resulted in 0.2% final SDS concentration for all samples and except for human tissue samples which had a final concentration of 0.6% SDS.

Antigen incubation was performed for 2 h at room temperature at 600 rpm on a microplate mixer (USA Scientific, 8182–2019). After three wash steps with TBS-T, 30 µl of detecting antibodies (total mouse PRKN antibodies, all 1 µg/ml final concentration) were added in blocking buffer to each well and incubated again for 2 h. Fifty µl of Sulfotag-labeled goat anti-mouse antibody (Meso Scale Diagnostics, R32AC-1; 1:500) was next added in blocking buffer and incubated for 1 h. After three additional wash steps, 150 µl MSD GOLD Read Buffer (Meso Scale Diagnostics, R92TG-2) was added to each well and plates were read on a MESO QuickPlex SQ 120 reader (Meso Scale Diagnostics, Rockville, MD, USA).

### Determining the limit of blank (LoB), limit of detection (LoD) and limit of quantification (LoQ)

The LoB, LoD, and LoQ were calculated using MSD-ECL values from recombinant PRKN and p-S65-PRKN according to *Armbruster and Pry* [57]. In brief, the LoBs were determined using the formula LoB = mean _blank_ +1.645(SD _blank_) where blocking buffer served as blank. The LoD was determined using serial dilutions of PRKN and p-S65-PRKN analytes and calculated with the following formula: LoD = LoB +1.645(SD _low concentration sample_). The LoQ was determined as the lowest concentration of analyte that could be measured with a predefined goal for imprecision (here under 10%) or is clearly distinguishable from the LoB with more than three times the standard deviation.

### Statistical analysis

Data analysis was performed in GraphPad Prism version 10 software. One-way ANOVA followed by Tukey’s multiple comparison test was used for the HEK293E cell and mouse data analysis. A Mann-Whitney U test was used for human brain analysis for two groups, while a Kruskal-Wallis test combined with Dunn’s test to correct for multiple comparisons was used for more than two groups, respectively.

## Supplementary Material

PRKN_ELISA_Supplement R2.docx

## Data Availability

The authors confirm that the data supporting the findings of this study are available within the article and/or its supplementary materials.
