# Supplementary material for: Robust and sensitive ELISA detection of total and activated PRKN
Source: Autophagy. 2026 Jul 3;22(11):3014–29. doi: 10.1080/15548627.2026.2694658 (PMC13712040; doi:10.1080/15548627.2026.2694658)
Supplement: Supplementary file 1 [file KAUP_A_2694658_SM6142.docx]

**SUPPLEMENTARY INFORMATION**

**Robust and Sensitive ELISA Detection of Total and Activated PRKN**

Jens O. Watzlawik^1^, Bernardo A. Bustillos^1^, Claudia Rodriguez Martinez^1^, Dennis W. Dickson^1,2^, Zbigniew K. Wszolek^3^, Owen A. Ross^1,2^, Wolfdieter Springer^1,2 #^, Fabienne C. Fiesel^1,2 #^

^1^ Mayo Clinic, Department of Neuroscience, Jacksonville, FL

^2^ Neuroscience PhD Program, Mayo Graduate School of Biomedical Sciences, Mayo Clinic, Jacksonville, FL, USA

^3^ Mayo Clinic, Department of Neurology, Jacksonville, FL

^#^ Corresponding authors:

Wolfdieter Springer, PhD: [Springer.Wolfdieter@mayo.edu](mailto:Springer.Wolfdieter@mayo.edu)

Fabienne C. Fiesel, PhD: [Fiesel.Fabienne@mayo.edu](mailto:Fiesel.Fabienne@mayo.edu)

**SUPPLEMENTARY FIGURES AND LEGENDS**

**Figure S1.** Schematic overview of PRKN protein with domain structure and epitope regions of PRKN antibodies. (**A**) Individual PRKN domains are color-coded, and amino acid (aa) numbers underneath indicate their respective start/end. Position of Ser65, which is phosphorylated by PINK1, and location of rare (R275W) and common (D394N) variants are shown on top. (**B**) Estimated or published binding epitopes of 15 total PRKN antibodies are shown as lines relative to the domain structure with colors indicating their host species (pink = rabbit, green = sheep, blue = mouse, yellow = goat). UBL – ubiquitin-like domain; R0-R2 – RING0-2; IBR – In-Between-RING; REP – repressor domain. (**C**) Mouse and human PRKN protein sequence alignment**.** Black asterisks indicate identical amino acids whereas mismatches between both species are indicated in pink. Seventy-six mismatches are present throughout the entire protein with 23 mismatches (30%) alone in the first 100 amino acids, which corresponds to the binding region for antibodies #1, #2, #5, #8, #9 and #13.

**Figure S2.** Heat-denaturing conditions result in higher PRKN MSD-ECL signals and higher signal-noise ratios using recombinant protein and HEK293E cells. (**A)** Recombinant total PRKN and p-S65-PRKN protein (range 100 fg/ml to 1 µg/ml) were either dissolved in RIPA without heat (non-heat-denatured) or in RIPA+2% SDS and heated for 5 minutes at 95°C (heat-denatured). All recombinant proteins were subsequently 10-fold diluted in blocking buffer before loading and then assessed in the PRKN ELISA (#3/#13). (**B)** WT, *PRKN* KO and *PINK1* KO HEK293E cells were treated with 20 µM CCCP for 0 or 1 h and either lysed in RIPA without heat or in RIPA plus 2% SDS and heated for 5 min at 95°C. All HEK293E cell lysates (30 µg total protein per well) were subsequently 10-fold diluted in blocking buffer and assessed in the total PRKN ELISA (#3/#13) showing the mean plus SD. MSD-ECL – Meso Scale Discovery-Electrochemiluminescence.

**Figure S3**. p-S65-PRKN ELISA specificity and testing of heat-denaturing conditions using recombinant protein and HEK293E cells. (**A**) Recombinant p-S65-/total PRKN and recombinant p-S65-Ub monomer, K48- and K63-linked p-S65-Ub tetramers (range 0-100 ng/ml), respectively, were used to coat the plate and p-S65-PRKN antibody #A was used for detection in a direct ELISA setting. (**B**) WT, *PRKN* KO and *PINK1* KO HEK293E cells were treated with 20 µM CCCP for 0 or 1 h and either lysed in RIPA without heat or in RIPA plus 2% SDS and heated for 5 minutes at 95°C. All HEK293E cell lysates (30 µg total protein per well) were subsequently 10-fold diluted in blocking buffer and assessed in the p-S65-PRKN ELISA (#A/#11). Shown is the mean ± SD. MSD-ECL – Meso Scale Discovery-Electrochemiluminescence; SD – standard deviation.

**Figure S4.** Determining optimal blocking buffer conditions for p-S65-PRKN and total PRKN ELISA. p-S65-PRKN (antibody pair #A/#11) and total PRKN ELISA (antibody pair #1/#11) were used in combination with recombinant p-S65-PRKN protein diluted in either blocking buffer or in *PRKN* KO HEK293E cell culture lysates. All antibodies were used in a concentration of 1 µg/ml. MSD-ECL – Meso Scale Discovery-Electrochemiluminescence.

**Figure S5.** p-S65-PRKN and total PRKN ELISA levels in PD patient fibroblasts with PRKN^R275W/Q^. The two promising p-S65-PRKN ELISA antibody combinations (#A/#11; #A/#13) and total PRKN ELISA antibodies were used to detect pS65-Ub and total PRKN levels in fibroblasts with compound heterozygous PRKN^R275W/Q^ mutation. Control cells without mutation and cells from a carrier with homozygous PINK1^Q456X^ mutation, which we previously have validated to cause the loss of PINK1 protein [30,54,56], were used as control. Cells were treated for 0, 4 and 8 h with mitochondrial depolarizer valinomycin (1 µM final concentration). Shown is the mean ± SD. MSD-ECL from two experiments – Meso Scale Discovery-Electrochemiluminescence; SD – standard deviation.

**Figure S6.** PRKN antibody comparison from different vendors respective in engineered vs. non-engineered format for p-S65-PRKN ELISA and/or total PRKN ELISA. (**A**) The total PRKN ELISAs utilizing capture antibody #1 was validated using PRK8 detecting antibodies from different vendors as indicated. Recombinant total PRKN served as antigen in different concentrations (range 0-100 ng/ml). Similarly, PRK8 detecting antibodies were tested in the p-S65-PRKN ELISA and combined with p-S65-PRKN antibody #A as capture agent. Recombinant p-S65-PRKN was used as antigen in a range of 0-100 ng/ml. All capture and detecting antibodies in both total PRKN and p-S65-PRKN ELISA were used in a concentration of 1 µg/ml. (**B**) PRKN antibody clone 21H24L9 was tested in standard vs. engineered formulation in the total PRKN ELISA when combined with PRKN-detecting antibody (Millipore, MAB5512; PRK8). All antibodies were used in a concentration of 1 µg/ml. Recombinant p-S65-PRKN protein was used in a concentration range from 0-100 ng/ml with log pg/ml concentration shown for better comparison. While the overall MSD-ECL signal range increased ~1.5-2-fold with the engineered clone across the tested concentrations, the background noise increased 4.5-fold as well without changing the assays limit of quantification (LoQ). MSD-ECL – Meso Scale Discovery-Electrochemiluminescence.
